# Microplastic exposure elicits sex-specific atherosclerosis development in lean low-density lipoprotein receptor-deficient mice

**DOI:** 10.1016/j.envint.2025.109938

**Published:** 2025-11-17

**Authors:** Ting-An Lin, Jianfei Pan, Mya Nguyen, Qianyi Ma, Liang Sun, Sijie Tang, Matthew J. Campen, Hong Chen, Changcheng Zhou

**Affiliations:** a Division of Biomedical Sciences, School of Medicine, University of California, Riverside, CA, USA; b Environmental Toxicology Graduate Program, University of California, Riverside, CA, USA; c Research Informatics, Department of Information Technology, Boston Children’s Hospital, Boston, MA, USA; d Department of Pharmaceutical Sciences, College of Pharmacy, University of New Mexico Health Sciences, Albuquerque, NM, USA; e Vascular Biology Program, Boston Children’s Hospital and Harvard Medical School, Boston, MA, USA

**Keywords:** Microplastics, Atherosclerosis, Cardiovascular disease, Single-cell RNA sequencing, Endothelial cell dysfunction

## Abstract

Microplastics (MPs) are small plastic particles emerging as significant environmental pollutants and humans are ubiquitously exposed to MPs. MPs can be detected in human atherosclerotic plaques and are associated with a higher risk of cardiovascular disease (CVD) and stroke in humans. However, the impact of MP exposure on the cardiovascular system remains elusive. In the current study, we investigated the effects of exposure to MPs at an environmentally relevant dose on atherosclerosis development in male and female low-density lipoprotein receptor-deficient (LDLR^−/−^) mice. LDLR^−/−^ mice were fed a semisynthetic low-fat (4.3 %), low-cholesterol (0.02 %) diet and exposed to 10 mg/kg body weight MPs via daily oral gavage for 9 weeks. Male and female LDLR^−/−^ mice fed the low-fat diet did not develop obesity phenotype and exposure to MPs did not affect adiposity and circulating lipid profiles in those lean mice. Intriguingly, MP exposure increased atherosclerotic lesion areas in the aortic root by 63 % (*p* = 0.0185) and brachiocephalic artery by 624 % (*p* = 0.0541) in male LDLR^−/−^ mice but did not significantly affect atherosclerosis in female mice. Single-cell RNA sequencing analysis of the whole aorta revealed that exposure to MPs affected the proportions and cellular processes of key atherogenesis-related cell types, especially endothelia cells. Consistently, MP exposure induced pro-atherogenic gene expression in murine primary endothelia cells and human endothelial cells in vitro. Our findings reveal the sex-specific atherogenic effects of MPs in vivo and provide mechanistic insights and new understanding of the impact of MPs on atherosclerosis development and CVD risk in humans.

## 1. Introduction

Microplastics (MPs) and nanoplastics (NPs) are emerging environmental pollutants due to the extensive use of plastics worldwide (Prata et al., 2020; Wu et al., 2022). The ubiquity of plastic particles leads to severe environmental pollution, which can be detected in aquatic, terrestrial, and atmospheric environments (Duis and Coors 2016; Richard et al., 2024; Ten Hietbrink et al., 2025). The widespread MPs and NPs can further migrate into food materials, causing contamination in drinking water, seasoning, vegetables, grains, meat, and seafoods (Sohail et al., 2023; Vitali et al., 2023). The contaminated food chain then contributes to the trophic transfer of plastics particles, exhibiting bioaccumulation and biomagnification manners to the exposed individuals (Batista et al., 2022; Carbery et al., 2018; Nelms et al., 2018). Humans are ubiquitously exposed to MPs and NPs through oral intake, inhalation, and dermal contact (Ramsperger et al., 2023; Sangkham et al., 2022). Studies have assessed that the intake of plastic particles ranges from 39,000 to 52,000 particles/person/year, and the consumption of oral intake and inhaled MPs ranges from 74,000 to 121,000 particles/person/year, suggesting that humans may be frequently exposed to MPs and NPs (Cox et al., 2019; Cox et al., 2020). MPs and NPs can be detected in human circulatory system and all major organs including brain, liver, intestine, stomach, spleen, kidney, lung, testis, placental, and blood vessel (da Silva Brito et al., 2022; Garcia et al., 2024; Hu et al., 2024; Leslie et al., 2022; Nihart et al., 2025; Yee et al., 2021).

Since MPs/NPs can be distributed through blood circulation (Amato-Lourenço et al., 2021; Liu and You 2023), bioaccumulation of MPs/NPs has been detected in human cardiovascular systems including atherosclerotic plaques, indicating a potential link between MP/NP exposure and cardiovascular disease (CVD) risk (Goldsworthy et al., 2025; Landrigan 2024; Liu et al., 2024; Makwana et al., 2025; Marfella et al., 2024; Prattichizzo et al., 2024). Despite advanced diagnosis and treatment, atherosclerotic CVD remains the leading cause of death world-wide (Lin and Zhou 2025; Roth et al., 2019; Vaduganathan et al., 2022). Atherosclerosis is characterized by the accumulation of lipids, leukocytes, and fibroblasts within the arterial wall (Libby 2021). A recent study has detected MPs/NPs in human carotid artery plaques, and the presence of MPs/NPs was associated with increased CVD events in patients (Landrigan 2024; Marfella et al., 2024). Another human study revealed that MP concentrations in carotid and coronary arteries containing atherosclerotic plaques were significantly higher than that in arteries without plaques (Liu et al., 2024). Interestingly, marine MP levels were significantly associated with the prevalence of cardiometabolic diseases including coronary artery disease in coastal counties of the United States (Makwana et al., 2025). Furthermore, blood MP concentrations have been associated with elevated atherosclerotic risk and increased complexity of vascular pathologies in acute coronary syndrome patients (Yang et al., 2024). These findings suggest that exposure to MPs may directly contribute to atherosclerosis development in humans, but the underlying mechanisms remain elusive.

Several animal studies previously investigated the impact of exposure to MPs or NPs on atherosclerosis in male apolipoprotein E-deficient (ApoE^−/−^) mice (Wang et al., 2023a; Wen et al., 2024a; Yang et al., 2025; Zhao et al., 2024; Zhong et al., 2024). However, most of those studies used the high-fat feeding conditions (Wang et al., 2023a; Wen et al., 2024a; Yang et al., 2025; Zhong et al., 2024). ApoE^−/−^ mice can develop obesity and diabetic phenotypes when fed high-fed diets, which may influence atherosclerosis development. In addition, those studies only used male mice but sex differences in atherosclerosis or CVD have been well-recognized in human and animal studies (Nour et al., 2023; Robinet et al., 2018; Sakkers et al., 2023; Vaura et al., 2022). Thus, it is not clear whether MP or NP exposures can affect atherosclerosis development in female ApoE^−/−^ mice.

In the current study, we used both male and female atherosclerosis-prone low-density lipoprotein receptor-deficient (LDLR^−/−^) mouse model under the low-fat diet feeding condition to investigate the impact of chronic MP exposure on atherosclerosis development. We selected LDLR^−/−^ mice because they serve as a well-established model for studying atherosclerosis. In addition, LDLR is important for lipoprotein homeostasis but does not have multitude of functions as ApoE (Getz and Reardon 2006). We also chose a semisynthetic low-fat, low-cholesterol AIN76 diet containing 4.3 % fat and 0.02 % cholesterol which has been successfully used to induce atherosclerosis without eliciting obesity and associated metabolic disorders in LDLR^−/−^ or ApoE^−/−^ mice (Hernandez et al., 2024; Hernandez et al., 2023; Liu et al., 2022; Meng et al., 2022; Sui et al., 2020; Sui et al., 2014; Sui et al., 2011; Teupser et al., 2003). We showed here that chronic MP exposure led to significantly increased atherosclerosis in lean male but not female LDLR^−/−^ mice without altering adiposity and circulating lipid profiles. We then conducted single cell RNA sequencing (scRNA-seq) analysis of the whole aorta to unveil MP-elicited changes of proportions and cellular processes of key cell types involved in atherosclerosis, especially endothelia cells (ECs). Further, MP exposure induced pro-atherogenic gene expression in murine primary ECs and human ECs in vitro. Our findings reveal the sex-specific atherogenic effects of MPs in vivo and provide novel mechanistic insights into MP-elicited CVD risk in humans.

## 2. Materials and methods

### 2.1. Animals and treatment

Micro-sized polystyrenes with 1.1 μm diameter (LB11) were purchased from MilliporteSigma. The red-fluorescent polystyrene MPs with 1 μm diameter were purchased from the Magsphere (PSF-001UM). To investigate the effects of MP exposure on atherosclerosis, development, we used atherosclerosis-prone LDLR^−/−^ mouse model (The Jackson Laboratories). Four-week-old male and female LDLR^−/−^ mice were fed a semisynthetic low-fat, low-cholesterol AIN76 diet (4.2 % fat and 0.02 % cholesterol; Research Diet) (Hernandez et al., 2024; Hernandez et al., 2023; Teupser et al., 2003) and were treated with 10 mg/kg body weight of MPs or vehicle control (ultrapure water) daily by oral gavage for 9 weeks. The selected 10 mg/kg body weight dose in our current study was based on previous animal studies and environmentally relevant doses (Deng et al., 2022; Leslie et al., 2022; Wang et al., 2023a; Wen et al., 2024a; Zhong et al., 2024). For the MP distribution experiment, red-fluorescent MPs (RF-MPs) were administered to 4-week-old male LDLR^−/−^ mice under the same conditions as unlabeled MPs. All mice were kept in pathogen-free microisolator cages in a temperature-controlled room (21 °C) with 12-hour light/dark cycle. On the day of euthanasia, mice were fasted for 6 h following the dark cycle, and blood and major organs were collected as previously described (Hernandez et al., 2024; Liu et al., 2022; Sui et al., 2014). All animal studies were performed in compliance with the approved IACUC protocols in University of California, Riverside.

### 2.2. Metabolic phenotype and blood analysis

Body weights were measured weekly, and the body leans and fat mass were measured by NMR spectroscopy (EchoMRI, Echo Medical System) on the day of euthanasia (Helsley et al., 2016; Hernandez et al., 2023; Liu et al., 2023). Serum total cholesterol and total triglyceride concentrations were measured using the Wako Cholesterol E enzymatic colorimetric assay (Wako, 999–02601) and the Wako L-type TG M assay (Wako, 994–02891) as previously described (Hernandez et al., 2023; Sui et al., 2021). The lipoprotein fractions were isolated in a Beckman Coulter XPN100-IVD ultracentrifuge as previously described (Hernandez et al., 2024; Hernandez et al., 2023; Meng et al., 2019; Sui et al., 2014).

### 2.3. Atherosclerotic lesion analysis

Atherosclerotic lesions were analyzed at the aortic root and brachiocephalic artery (BCA) of LDLR^−/−^ mice as we previously described (Hernandez et al., 2024; Liu et al., 2022). The collected hearts and BCAs were embedded in optimal cutting temperature (OCT) compound. The hearts were sectioned at a 12 μm thickness with three valves of aortic root being presented in the same plane as aortic root, and the BCAs were sectioned at a 10 μm thickness from distal to proximal. The sections were fixed with 4 % paraformaldehyde (PFA) for 15 min, rinsed with PBS for 15 min, and stained with Oil Red O for 1 h. After staining, the slides were rinsed again with PBS for 15 min. Atherosclerotic lesions were then quantified in three equidistant stained sections 200, 400 and 600 μm proximal from the branching point of the brachiocephalic artery into the carotid and subclavian arteries (Sui et al., 2020; Wang et al., 2018).

### 2.4. Immunofluorescence staining

The sections of mouse aortic root were used for immunofluorescence staining (Hernandez et al., 2024; Wang et al., 2018). The slides were first fixed with 4 % PFA and washed by PBS containing 0.1 % Triton X-100 (PBST) for 15 min. The samples then incubated with 1:100 antibodies against MOMA-2 (Bio-Rad, MCA519a,) and ACTA2 (Abcam, ab5694, USA) at 4 °C overnight. After incubating with primary antibodies, slides were rinsed with PBST and then incubated with fluorescent 1:500 secondary antibodies (Invitrogen, USA). The nuclei were finally stained with 4’,6-diamidino-2-phenylindole (DAPI) (Vector Laboratories). Images were captured and measured by a Nikon fluorescence microscope.

### 2.5. Primary murine endothelial cell isolation and human endothelial cell culture

Murine primary endothelia cells (ECs) were isolated from the brain of male LDLR^−/−^ mice as we previously described (Rahman et al., 2016). Tissues were rinsed with PBS followed by ethanol and minced into small fragments in DMEM high glucose supplemented. The tissues then digested in 2 mg/mL collagenase I for 30 min and filtered by 20 μm cell strainer. Following digestion, the cell suspension was centrifuged and resuspended in MCDB131 medium supplemented with brain extract. Cells were then seeded into 6- or 96-well culture dishes and harvested. Human endothelial cell line, HMEC-1, was purchased from ATCC (CRL-3243) and cultured in MCDB131 completed medium (Hernandez et al., 2024; Hernandez et al., 2023; Pickering et al., 2019). For MP treatment, primary ECs and HMEC-1 cells were treated with MPs at concentrations of 0 and 10 mg/L for 24 h and prepared for RNA isolation or other assays.

### 2.6. Reactive oxygen species (ROS) assays

Detection of reactive oxygen species (ROS) was performed as previously described (Kim and Xue 2020). After MP treatment, human HMEC-1 ECs were washed once with PBS and incubated with 10 μM 2′,7′-dichlorodihydrofluorescein diacetate (H_2_DCFDA) (Thermo Fisher Scientific, D399) in serum-free MCDB131 medium at 37 °C for 30 min in the dark. After staining, the cells were washed three times with PBS, and fluorescence was measured (excitation = 495 nm, emission = 527 nm) using a BioTek Synergy H1 Multimode Reader (Agilent). Representative images were also captured by a Nikon fluorescence microscope.

### 2.7. RNA isolation and quantitative real-time PCR

Total RNA of primary ECs and HMEC-1 cells was isolated by TRIzol reagent (Thermo Fisher Scientific, USA). After RNA extraction, iScript^™^ Reverse Transcription Supermix (Bio-Rad) was applied for reverse transcription, and SYBR Green Supermix (Bio-Rad) was used for quantitative real-time PCR (QPCR) on a CFX Real-Time PCR Instrument (Bio-Rad) (Hernandez et al., 2024; Wang et al., 2023b). The primer sequences are listed in Supplemental Table 1.

### 2.8. Single-cell RNA sequencing analysis of aorta

Aortic sample preparation and library construction were conducted with enzymatic digestion protocol as previously described (Cui et al., 2023; Kalluri et al., 2019). Briefly, three aortas from control and MP-exposed mice were pooled, cut into small pieces and digested with enzyme solute to obtain single cell suspensions. The digested tissues were filtered through a 40 μm cell strainer, and the isolated aortic cells were counted and diluted into 1000 cells/μL. The scRNA-seq library construction was performed according to the 10x genomics protocol (GEMs, Chromium Fixed RNA Profiling Kit, 10x Genomics). The prepared libraries were sequenced on the HiSeq 2500 Sequencing System (Illumina, San Diego, CA) with 100-bp paired end reads.

The sequencing raw reads were mapped to the mouse reference genome (version mm10-2020-A) using Cell Ranger (version 8.0.1), generating three output files: barcodes, features, and matrix. We then removed ambient RNA using SoupX (version 1.6.2), created a Seurat object and performed quality control using the Seurat R package (version 4.2.3) and scds (version 1.14.0), filtering out low-quality cells, doublets, and cells with high mitochondrial content. Only high-quality cells expressing between 200 and 8,000 genes and containing fewer than 20 % mitochondrial reads, 20 % ribosomal, and 1.5 % hemoglobin genes were retained in the aortic datasets. In addition, genes expressed in fewer than three cells were excluded. After filtering, the high-quality data from control and MP-exposure mice were merged, normalized, scaled, followed by performed principal component analysis (PCA). Batch effects were then corrected using the Harmony (version 0.1.1) method (Korsunsky et al., 2019). After cell clustering, marker genes were used to annotate the specific cell types (Chakraborty et al., 2023). Cell type annotation was also consolidated by enrichR (version 3.1) using top 100 markers and by SingleR (version 2.0.0). Differences in cell type proportions between control and MP-exposure aortas were assessed using the scProportionTest (Miller et al., 2021). Trajectory analysis of different endothelial subclusters was performed by Monocle 2 (Qiu et al., 2017). All data associated with this study are present in the main text or the supplementary materials. The scRNA-seq data generated in this study have been deposited in the GEO database under accession code: GSE306246.

### 2.9. Statistical analysis

All data except the scRNA-seq data are presented as the mean ± SEM and n numbers are listed in the figure legends. Individual pairwise comparisons were analyzed between control and exposed groups by two-sample, two-tailed Mann-Whitney *U* test. The analysis was performed using GraphPad Prism, as *p* < 0.05 was considered statistically significant. Precise *p* values are also provided in the figures, figure legends, or main text.

## 3. Results

### 3.1. MP exposure does not affect body weight and adiposity in lean LDLR^−/−^ mice

**Fig. 1.**
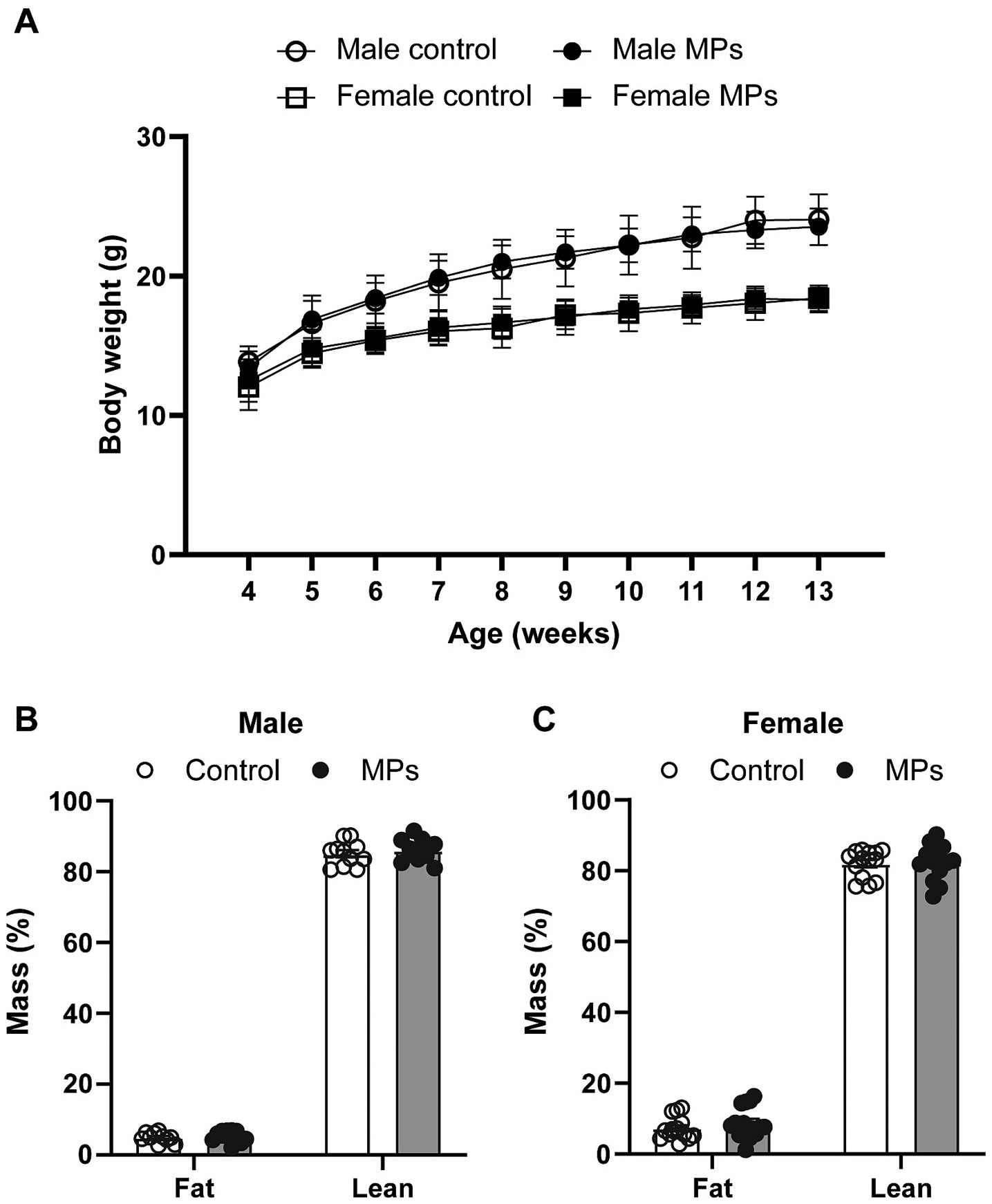
Microplastic exposure does not affect body weight and adiposity of male and female LDLR^−/−^ mice. Four-week-old male and female LDLR^−/−^ mice were fed a low-fat AIN76 diet and treated with 10 mg/kg body weight microplastics (MPs) or vehicle control by oral gavage daily for 9 weeks before euthanasia. (A) Growth curves of male and female LDLR^−/−^ mice. (B and C) Fat and lean mass of male (B) and female (C) LDLR^−/−^ mice. (n = 10–16; two-sample, two-tailed Mann-Whitney *U* test). All data are presented as mean ± SEM.

To evaluate the impact of MP exposure on atherosclerosis development, male and female LDLR^−/−^ mice were treated with 10 mg/kg body weight of MPs or vehicle control by daily oral gavage for 9 weeks. While many studies used high-fat diets to induce severe hyperlipidemia and atherosclerosis development in LDLR^−/−^ or ApoE^−/−^ mice, those mice also developed diet-induced obesity and diabetic phenotypes, which may indirectly influence atherosclerosis development. Thus, we fed the LDLR^−/−^ mice a low-fat, low-cholesterol AIN76 diet containing 4.3 % fat and 0.02 % cholesterol (Hernandez et al., 2024; Hernandez et al., 2023; Teupser et al., 2003). This diet has been successfully used by us and others to induce atherosclerosis without eliciting obesity and associated metabolic disorders in LDLR-deficient or ApoE-deficient mice (Hernandez et al., 2024; Hernandez et al., 2023; Liu et al., 2022; Meng et al., 2022; Sui et al., 2020; Sui et al., 2014; Sui et al., 2011; Teupser et al., 2003). Exposure to MPs did not affect body weight and growth curve of male and female LDLR^−/−^ mice (Fig. 1). Consistent with our previous studies (Hernandez et al., 2024; Hernandez et al., 2023; Liu et al., 2022; Meng et al., 2022), LDLR^−/−^ mice fed this low-fat AIN76 diet did not develop obesity phenotype (Figs. 1B and C). MP- or vehicle control-exposed mice also had similar body composition, including fat mass and lean mass (Figs. 1B and C). Thus, chronic MP exposure did not affect the body weight and adiposity in lean male and female LDLR^−/−^ mice.

### 3.2. MP exposure leads to sex-specific atherosclerosis exacerbation in LDLR^−/−^ mice without altering serum lipid profiles

**Fig. 2.**
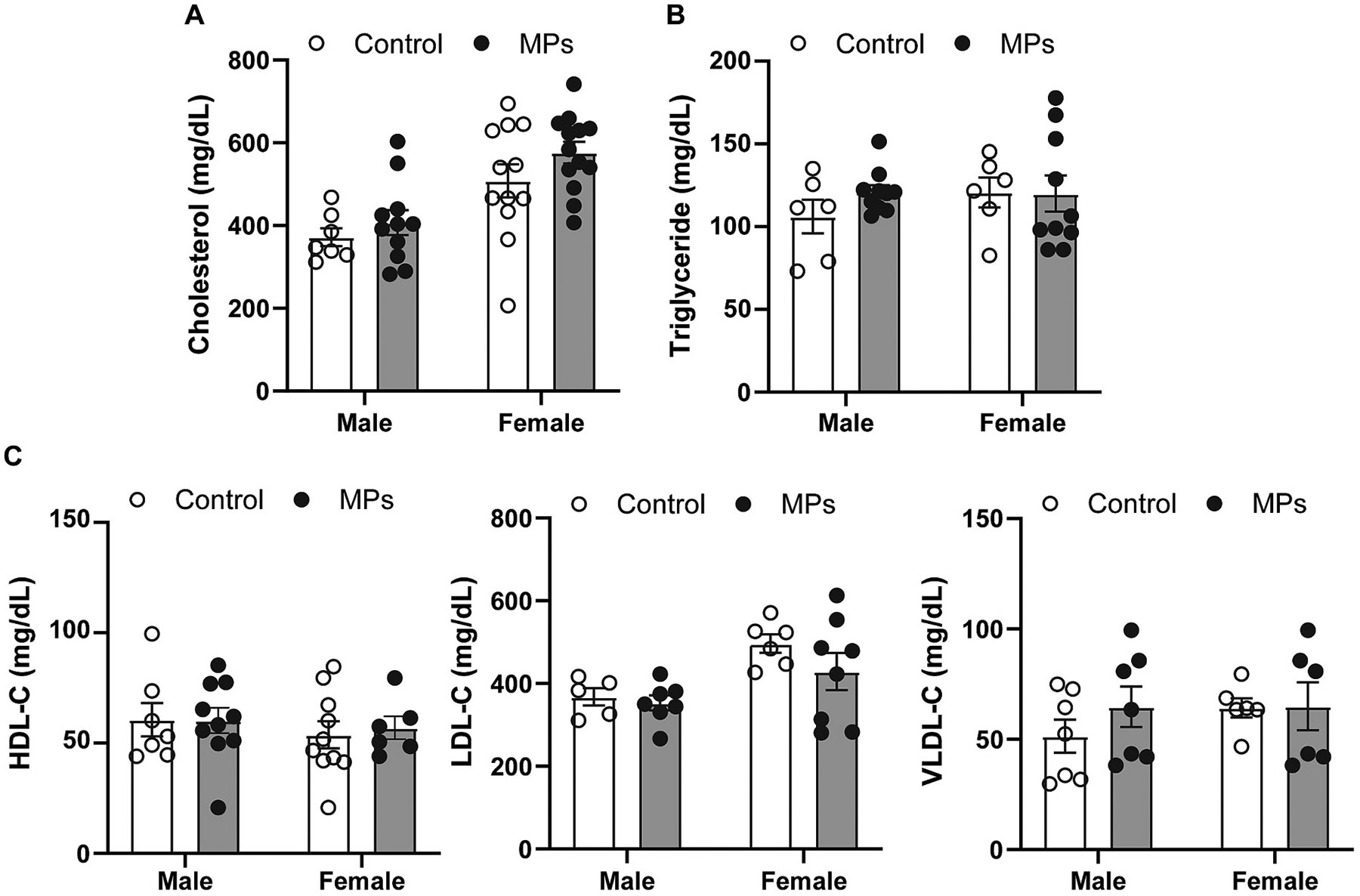
Microplastic exposure does not alter serum lipid profiles in lean LDLR^−/−^ mice. Four-week-old male and female LDLR^−/−^ mice were fed a low-fat AIN76 diet and treated with 10 mg/kg body weight microplastics (MPs) or vehicle control by oral gavage daily for 9 weeks before euthanasia. (A and B) Serum cholesterol (A) and triglyceride (B) levels in male and female LDLR^−/−^ mice were measured (n = 6–13; two-sample, two-tailed Mann-Whitney *U* test). (C) Lipoprotein fractions (VLDL, LDL, and HDL) were isolated from serum, and the cholesterol levels of each fraction were measured (n = 5–10; two-sample, two-tailed Mann-Whitney *U* test). All data are presented as mean ± SEM. HDL, high-density lipoprotein; LDL, low-density lipoprotein; VLDL, very low-density lipoprotein.

We next examined serum lipid levels and found that exposure to MPs did not affect serum cholesterol and triglyceride levels in both male and female LDLR^−/−^ mice (Figs. 2A and B). Serum lipoprotein fractions were then separated, and MP exposure did not affect VLDL, LDL, and HDL cholesterol levels (Fig. 2C).

**Fig. 3.**
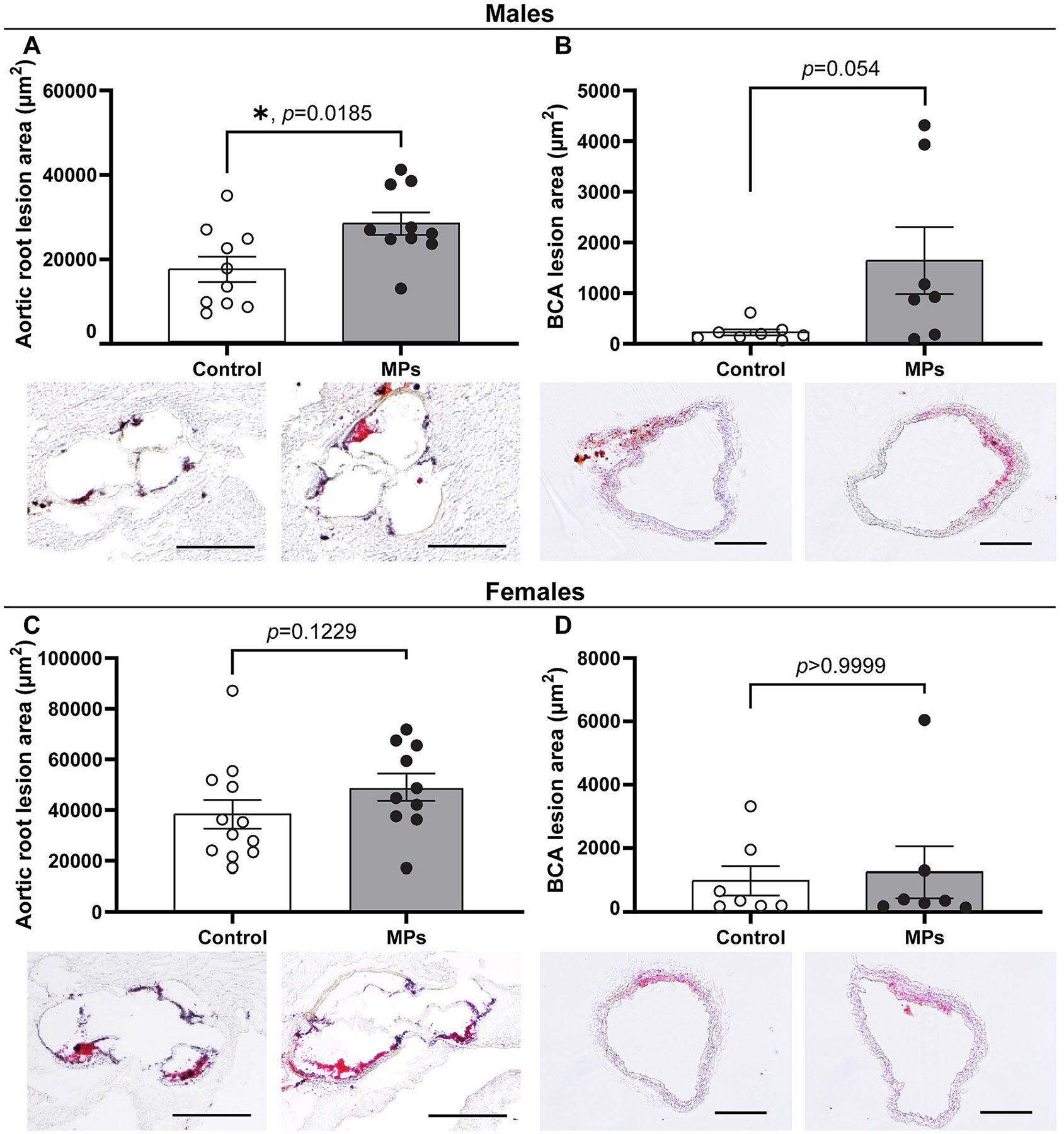
Exposure to microplastics leads to sex-specific atherosclerosis exacerbation in LDLR^−/−^ mice. Four-week-old male and female LDLR^−/−^ mice were fed a low-fat AIN76 diet and treated with 10 mg/kg body weight microplastics (MPs) or vehicle control by oral gavage daily for 9 weeks before euthanasia. Quantitative analysis of lesion area in the aortic root (A and C) and brachiocephalic artery (B and D) of male (A and B) and female (C and D) LDLR^−/−^ mice (n = 7–12; * *p* < 0.05; two sample, two-tailed Mann-Whitney *U* test). Representative Oil Red O–stained sections are shown below the quantification data (A and C, scale bar = 500 μm; B and D, scale bar = 200 μm). All data are presented as mean ± SEM. The mean values and precise *p* values are also provided in Supplemental Table 2.

Atherosclerotic lesion areas were then analyzed in the aortic root and brachiocephalic artery (BCA) of male and female LDLR^−/−^ mice. Despite of unchanged lipid profiles, exposure to MPs led to significantly increased atherosclerotic lesion areas by 63 % in the aortic root of male LDLR^−/−^ mice (27,979 ± 8,467 μm^2^ vs. 17,157 ± 9,434 μm^2^; 1.63-fold; *p* = 0.0185) (Fig. 3A and Supplemental Table 2). Consistently, exposure to MPs also increased the atherosclerotic lesion areas in the BCA of male LDLR^−/−^ mice by 624 % (1,643 ± 1,743 μm^2^ vs. 227 ± 170 μm^2^; 7.24-fold; *p* = 0.0541) (Fig. 3B and Supplemental Table 2). By contrast, MP exposure did not significantly affect the atherosclerotic lesion areas in the aortic root (49,096 ± 17,030 μm^2^ vs. 38,341 ± 19,791 μm^2^; 1.28-fold; *p* = 0.1229) or BCA (1,222 ± 2,153 μm^2^ vs. 957 ± 1,208 μm^2^; 1.28-fold; *p* > 0.9999) in female LDLR^−/−^ mice (Figs. 3C and D, Supplemental Table 2). We also evaluated the atherosclerotic plaque composition of male LDLR^−/−^ mice. MP exposure did not affect the smooth muscle cell (SMC) contents but significantly increased macrophage contents in the atherosclerotic plaques of male LDLR^−/−^ mice (Supplemental Fig. 1).

**Fig. 4.**
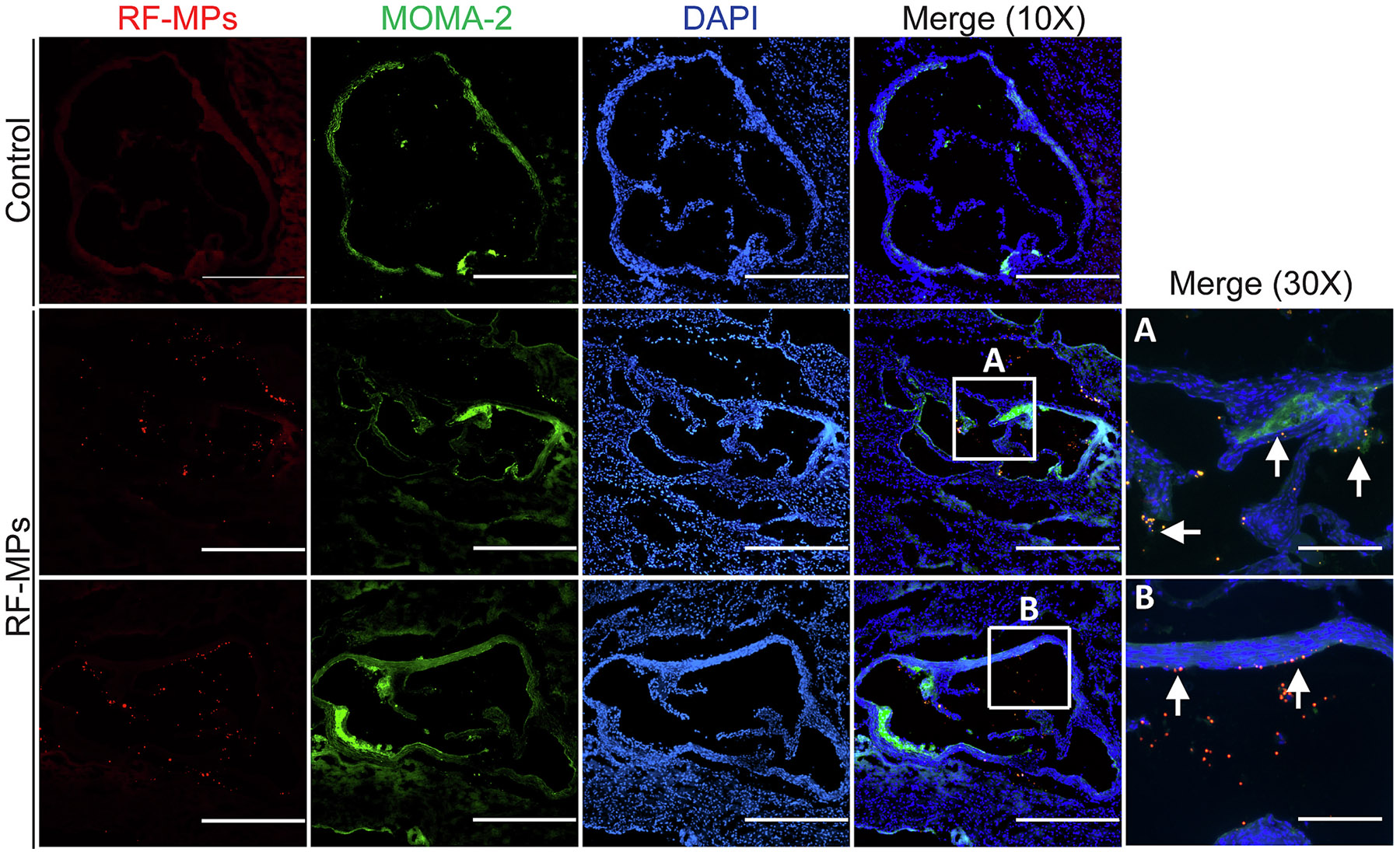
The distribution of red-fluorescent microplastics in the aortic root of LDLR^−/−^ mice. Four-week-old male LDLR^−/−^ mice were fed a low-fat AIN76 diet and treated with 10 mg/kg body weight red fluorescent MPs (RF-MPs) or vehicle control (Control) daily by oral gavage for 9 weeks before euthanasia. Representative images of immunofluorescence staining of macrophage marker MOMA-2 (green) and RF-MPs (red) in the aortic root of LDLR^−/−^ mice (scale bar = 500 μm for 10X image; 200 μm for 30X image). The nuclei were stained with DAPI (blue).

To determine whether MPs can indeed enter atherosclerotic plaques or vascular tissues, we exposed male LDLR^−/−^ mice with 10 mg/kg body weight of red-fluorescent MPs (RF-MPs) for 9 weeks under the same feeding condition. RF-MPs can indeed be detected within the atherosclerotic lesions of LDLR^−/−^ mice (Fig. 4). Further, RF-MPs can also locate in the intima of aortic vascular tissue (Fig. 4). Collectively, our result indicated that exposure to MPs did not affect circulating lipid profiles but led to sex-specific exacerbation of atherosclerosis in LDLR^−/−^ mice.

### 3.3. Single cell RNA sequencing analysis reveals the transcriptomic atlas in the aorta of LDLR^−/−^ mice in response to MP exposure

**Fig. 5.**
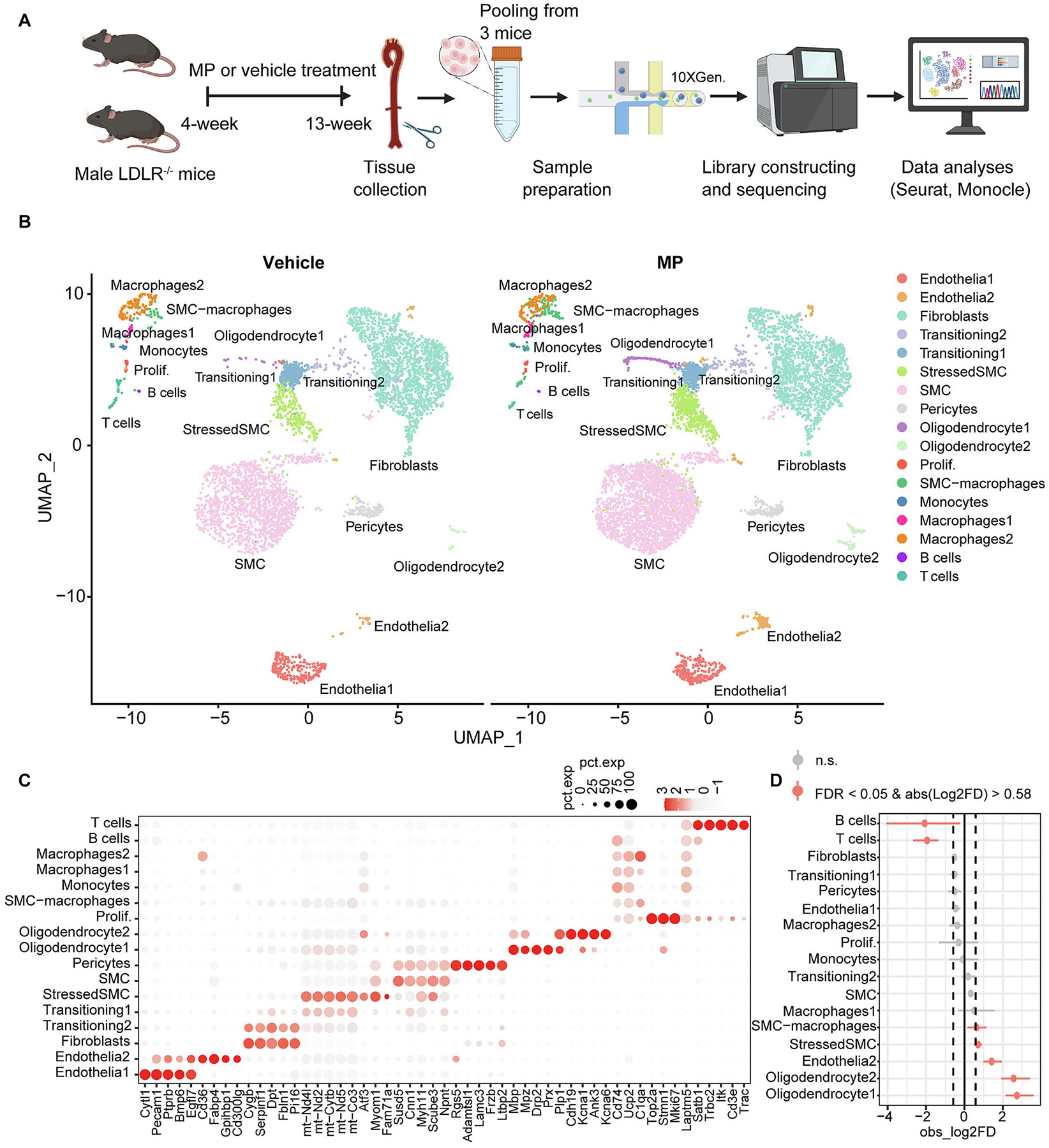
Single cell RNA sequencing analysis reveals the transcriptomic atlas in the aorta of LDLR^−/−^ mice in response to microplastic exposure. Four-week-old male LDLR^−/−^ mice were fed a low-fat AIN76 diet and treated with 10 mg/kg body weight microplastics (MPs) or vehicle control by oral gavage daily for 9 weeks before euthanasia. Single cell RNA sequencing (scRNA-seq) was performed to analyze the cellular heterogeneity in the aorta based on 10 × Genomics platform. (A) Schematic pipeline of scRNA-seq analysis in the aorta of male LDLR^−/−^ mice. (B) UMAP visualization of cell types present in the mouse aorta of MP-treated mice (MP) and control mice (Vehicle). All cell cluster identities are indicated in the figure. (C) Dot plot of the marker genes identified in each cell cluster. The size of each circle represents the fraction of cells in each cluster expressing at least one detected transcript of each gene. The color scale indicates the scaled average expression level. (D) Cell type proportion differences were assessed using scProportionTest for different cell types between control and MP-exposed samples. The x-axis denotes the fold difference in cell-type proportions (log2 scale). Points marked in red indicate clusters with significantly different proportions of cells between the two groups. Horizontal lines around the points indicate the confidence interval for the magnitude of difference for a specific cluster, calculated via bootstrapping.

To investigate the potential mechanisms for MP-elicited atherosclerosis, we applied scRNA-seq to analyze the transcriptomic changes in the aorta. Aortas of male LDLR^−/−^ mice exposed to vehicle control or MPs were collected and processed using the 10 × Genomics platform to obtain dissociated cells (Fig. 5A). A total of 17 cell clusters were identified and visualized in uniform manifold approximation and projection (UMAP), with cluster-specific marker genes displayed in a dot plot (Figs. 5B and C). To compare cell composition differences between the MP-exposed and control groups, we performed a single-cell proportion test to calculate *p*-values and confidence intervals. Interestingly, MP exposure significantly increased the composition of endothelia cells (EC), stressed SMCs, SMC-derived macrophages, and oligodendrocytes, while decreasing the proportions of B cells and T cells (Fig. 5D).

**Fig. 6.**
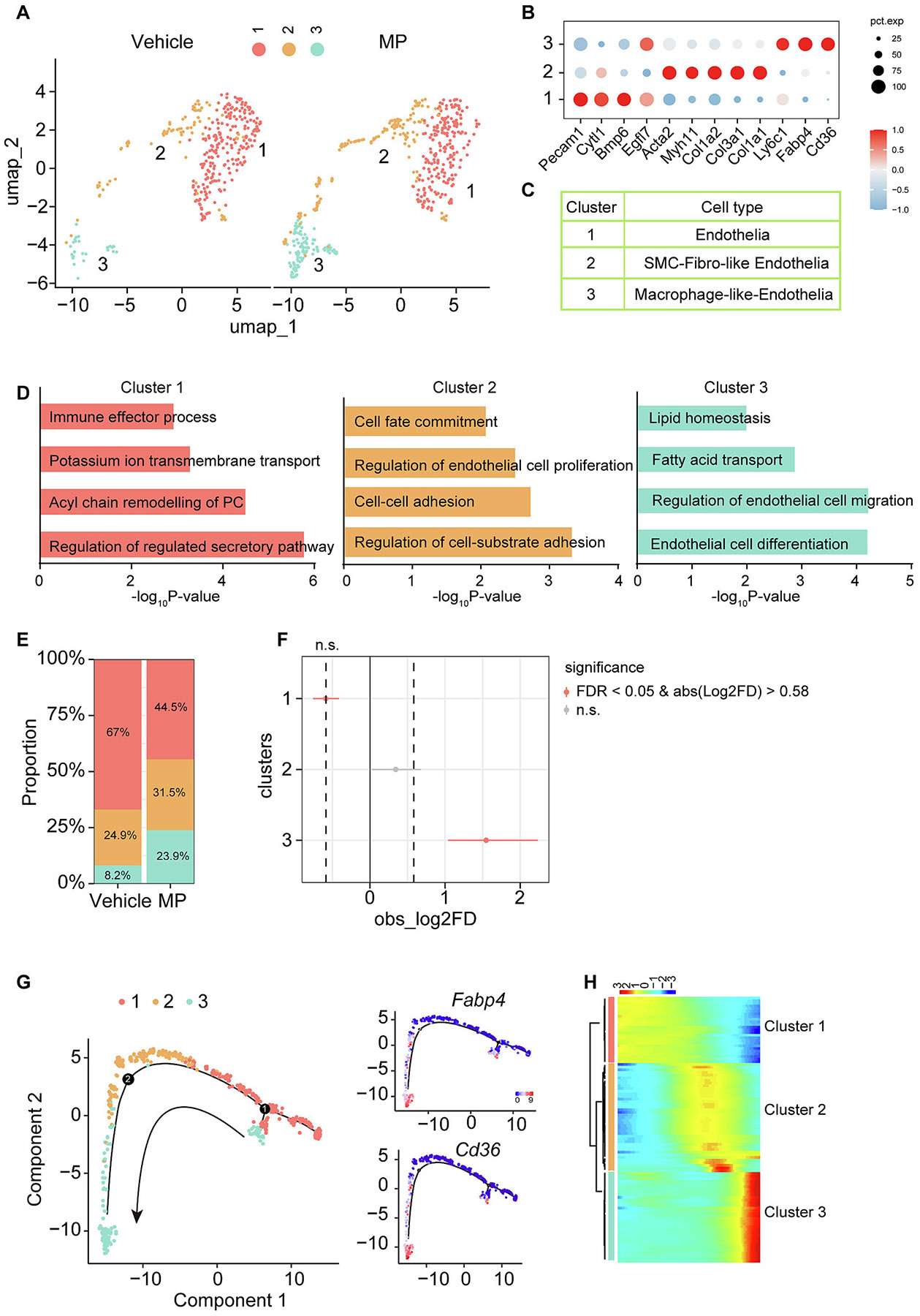
Exposure to microplastics affects different endothelial cell subclusters in the aorta of male LDLR^−/−^ mice. Four-week-old male LDLR^−/−^ mice were fed a low-fat AIN76 diet and treated with 10 mg/kg body weight microplastics (MPs) or vehicle control by oral gavage daily for 9 weeks before euthanasia. (A) UMAP visualization of aortic endothelia cells (ECs) from control and MP-exposed samples clustered into three distinct subclusters. (B) Dot plot of marker genes used to identify different aortic EC subclusters. (C) Cell types of different aortic EC subclusters. (D) Pathway analysis of Gene Ontology Biological Process (GOBP) terms for the highly expressed genes from each aortic EC subclusters. (E and F) Bar graph (E) and scProportionTest (F) analysis of cell type proportions for different aortic EC subclusters between control and MP-exposure samples. Statistical significance was defined as the FDR < 0.05 and fold change > 1.5. (G) Pseudotime analysis of the three aortic EC subclusters using Monocle 2. *Cd36* and *Fabp4* showed high expression levels in subcluster 3. (H) Pseudotime heatmap showing the top 100 differentially expressed genes across aortic EC subclusters along the pseudotime trajectory.

The increased proportion of ECs may represent wound healing and vascular repair in response to MP exposures. We next focused on the dynamic change in different EC clusters. For the UMAP analysis (Fig. 6A), the ECs were classified into 3 subclusters based on subclusterspecific marker genes (Figs. 6B and C). For example, EC subcluster 1 was identified as normal ECs which expressed *Pecam1*, *Cytl1*, and *Bmp6*; and EC subcluster 3 was identified as macrophage-like ECs, which expressed *Ly6c1*, *Fabp4*, and *Cd36* (Figs. 6B and C). Gene Ontology (GO) analysis showed that three different EC clusters have different enriched biological processes (Fig. 6D and Supplemental Table 3). For example, the enriched biological processes in EC subcluster 2 are associated with regulation of cell-substrate adhesion, cell–cell adhesion, and regulation of EC proliferation, and EC subcluster 3 had enriched biological processes such as regulation of EC differentiation, fatty acid transport, and lipid homeostasis (Fig. 6D).

Interestingly, MP exposure led to increased population of EC subcluster 3 but decreased EC subcluster 1 population (Figs. 6E and F). Combined with pseudotemporal trajectories and differentially expressed gene (DEG) analyses, we found that ECs had increased expression of pro-atherogenic genes including *Cd36* and *Fabp4* after MP exposure (Figs. 6G and H; Supplemental Table 3). Taken together, MP exposure may affect EC gene expression and functions to elicit atherogenic effects in vivo.

### 3.4. Exposure to MPs leads to dysregulated gene expression in murine primary endothelia cells and human endothelial cells in vitro

**Fig. 7.**
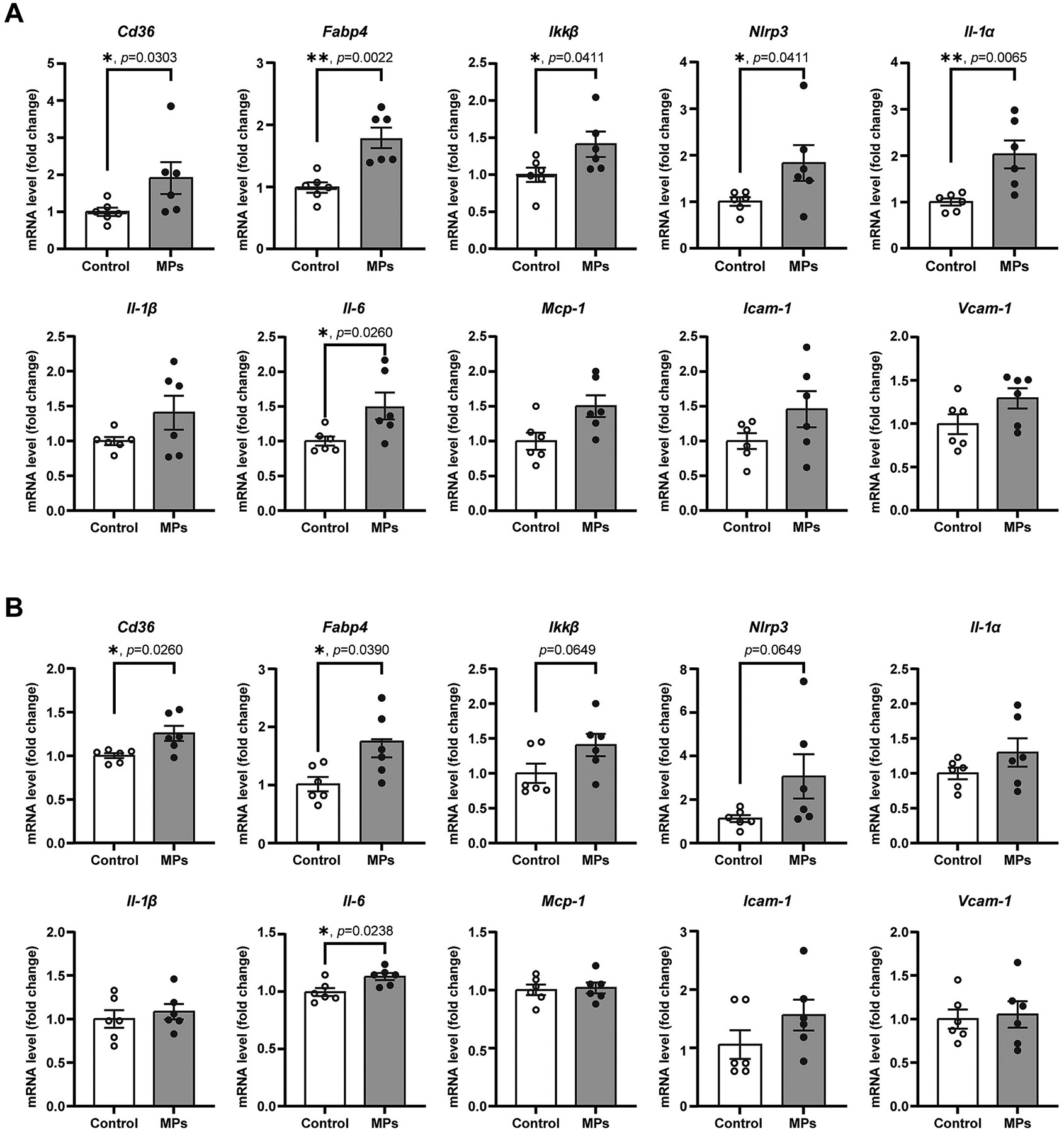
Microplastic exposure leads to dysregulated gene expression in murine primary endothelia cells and human endothelial cells in vitro. Murine primary endothelial cells (ECs) isolated from male LDLR^−/−^ mice and human HMEC-1 ECs were treated with vehicle control or 10 mg/L microplastics (MPs) for 24 h followed by total RNA extraction. The expression levels of indicated genes in ECs were analyzed by qPCR (n = 6; *, *p* < 0.05; **, *p* < 0.01; two sample, two-tailed Mann-Whitney *U* test). All Data are represented as mean ± SEM.

To elucidate the potential impact of MP exposure on EC function, we isolated primary ECs from male LDLR^−/−^ mice and treated them with MPs in vitro. Consistent with scRNA-seq results, MP treatment also led to increased expression of *Cd36* and *Fabp4* in ECs in vitro (Fig. 7). As inflammatory responses are the driving force of atherosclerosis development (Hernandez and Zhou 2021; Libby 2002; Sui et al., 2014), we also analyzed the expression of key proinflammatory genes in ECs. MP treatment stimulated the expression of several pro-atherogenic genes including *Il-1α, Il-6,* and *Nlrp3* (Fig. 7A). In addition, MP exposure also increased the expression levels of *Ikkβ* (Fig. 7A), a central coordinator of innate immunity and inflammation that has important function in atherogenesis (Hernandez and Zhou 2021; Park et al., 2012; Sui et al., 2014).

To elucidate the potential impact of MP exposure on human ECs, we treated human HMEC-1 ECs (Hernandez et al., 2024; Hernandez et al., 2023; Pickering et al., 2019) with the same dose of MPs. Consistently, MP exposure increased the expression of proatherogenic genes including *Cd36*, *Fabp4*, and *Il-6* in HMEC-1 cells (Fig. 7B). In addition, MP exposure also led to increased production of reactive oxygen species (ROS) in those cells (Supplementary Fig. 2), corresponding with increased pro-atherogenic gene expression. Collectively, these findings suggest that MP exposure can cause dysregulated EC gene expression and EC dysfunction, which may contribute to increased atherosclerosis in vivo.

## 4. Discussion

Due to their durability, versatility, and low cost, plastics are an essential part of modern society, and global plastic production has rapidly increased over the past century (Geyer et al., 2017). Humans are ubiquitously exposed to small plastic particles and increasing evidence suggests that exposure to these particles may affect development of chronic diseases including atherosclerotic CVD (Landrigan 2024; Liu et al., 2024; Marfella et al., 2024; Prattichizzo et al., 2024; Yang et al., 2024). However, the direct impact of exposure to MPs or NPs on atherosclerosis development is poorly understood. In this study, we investigated how exposure to MPs affects atherosclerosis development in lean LDLR^−/−^ mice. Interestingly, we found that MP exposure led to increased atherosclerosis in male but not female LDLR^−/−^ mice. We then conducted scRNA-seq analysis of aorta and revealed the impact of MP exposure on composition of several key cell populations required for atherogenesis. Further, exposure to MPs altered several EC subclusters and affected EC transcriptome. In vitro studies then showed the effects of MP treatment on pro-atherogenic gene expression in murine and human ECs. To the best of our knowledge, this is the first animal study to show the sex-specific atherogenic effects of MP exposure in LDLR^−/−^ mice.

Recent human studies have revealed the presence of MPs and NPs in the arteries and carotid artery plaque (Landrigan 2024; Liu et al., 2024; Marfella et al., 2024). Moreover, the increased MPs/NPs levels are associated with increased cardiovascular events of atherosclerotic plaque formation (Landrigan 2024; Liu et al., 2024; Marfella et al., 2024). Consistent with our results, several animal studies previously showed that exposure to MPs or NPs can lead to increased atherosclerosis in ApoE^−/−^ mice (Wang et al., 2023a; Wen et al., 2024a; Yang et al., 2025; Zhao et al., 2024; Zhong et al., 2024). For example, Zhao et al., recently showed that exposure to male ApoE^−/−^ mice 0.5 μm MPs via drinking water (1 μg/mL) for 20 weeks increased atherosclerotic lesion formation in those mice (Zhao et al., 2024). Several other studies also found MP or NP exposures led to increased atherosclerosis in male ApoE^−/−^ mice under HFD feeding conditions (Wang et al., 2023a; Wen et al., 2024a; Yang et al., 2025; Zhong et al., 2024). However, these studies only used male ApoE^−/−^ mice and it is not clear whether similar exposure conditions also affect atherosclerosis development in female ApoE^−/−^ mice. Furthermore, most groups used HFD for their atherosclerosis studies (Wang et al., 2023a; Wen et al., 2024a; Yang et al., 2025; Zhong et al., 2024). High-fat feeding-induced obesity and metabolic dysfunction may affect the atherosclerotic lesion development. Thus, it is possible that HFD-induced obesity and metabolic dysfunction served as the secondary pro-atherogenic factors that influence MP or NP-induced atherosclerosis development in those mice.

To avoid the additional stresses of obesity and metabolic dysfunctions presented in HFD-fed models, we fed the male and female LDLR^−/−^ mice a modified low-fat and low-cholesterol AIN76 diet (Teupser et al., 2003). We and others have used this AIN76 diet to successfully induce atherosclerosis in LDLR^−/−^ or ApoE^−/−^ mice without causing additional obesity and associated metabolic disorders in many studies (Hernandez et al., 2024; Hernandez et al., 2023; Liu et al., 2022; Meng et al., 2022; Rodriguez et al., 2013; Sui et al., 2020; Sui et al., 2014; Sui et al., 2011; Teupser et al., 2003; Wolfrum et al., 2007). Indeed, LDLR^−/−^ mice fed this diet did not develop obesity phenotype in the current study, and they were lean with normal adiposity. MP exposure did not affect the body weight or adiposity of these mice but still led to increased atherosclerosis in male but not female LDLR^−/−^ mice. Since exposure to MPs did not alter serum lipid profiles, MP-elicited atherosclerosis in male LDLR^−/−^ mice was unlikely affected by other secondary effects such as obesity and circulating cholesterol levels.

The selected 10 mg/kg body weight MP dose in our current study was based on previous animal studies and environmentally relevant doses (Deng et al., 2022; Wang et al., 2023a; Wen et al., 2024a; Zhong et al., 2024). For example, Wen et al., previously treated ApoE^−/−^ mice with 5 or 10 mg/kg body weight NPs for 3 months to study their atherogenic effects (Wen et al., 2024a). Wang et al., and Zhong et al., exposed ApoE^−/−^ mice with 2.5–250 mg/kg body weight of NPs for 19 weeks under high-fat feeding condition to study atherosclerosis development (Wang et al., 2023a; Zhong et al., 2024). Wang et al., also measured the blood NP levels after 24-hour exposure and they found that blood NP levels were distributed in the blood in a dose-dependent manner (Wang et al., 2023a). The blood NP levels of the three exposed doses (2.5, 25, and 250 mg/kg body weight) were 0.17, 0.51, and 3.37 μg/mL, respectively (Wang et al., 2023a). These values were within the range of reported plastic particle levels in human blood (1.6 ug/mL) (Leslie et al., 2022). In addition, another study estimated the mouse equivalent MP dose to be 2.7–8.7 mg/kg body weight based on the estimated daily intake of MPs (13 to 39.3 mg) for humans (Deng et al., 2022). Therefore, our selected dose is close to the estimated mouse equivalent MP exposure dose and is appropriate for studying the impact of MP exposure on atherosclerosis development in vivo. Nevertheless, we only selected a single dose for the MP exposure study, which limited our ability to assess dose-dependent effects of MP exposure on atherosclerosis. In addition, 9-week exposure period was also relatively short as compared to other studies (Wang et al., 2023a; Wen et al., 2024a; Yang et al., 2025; Zhao et al., 2024; Zhong et al., 2024). The lean LDLR^−/−^ mice fed the low-fat diet did not develop large and advanced lesions in our study. Thus, we could not assess the impact of longer-term MP exposures on atherosclerotic plaque complex and vulnerability. Those are the limitations of our study and future animal studies using more doses and longer exposure times should be considered to further investigate the impact of MP exposure on atherosclerosis development.

It is intriguing that MP exposure can lead to increased atherosclerosis in male but not female LDLR^−/−^ mice. Sex differences in CVD and its associated risk factors have been well documented in humans (Nour et al., 2023; Sakkers et al., 2023; Vaura et al., 2022). Sex differences have also been widely reported in mouse atherosclerosis studies (Robinet et al., 2018). However, the detailed mechanisms responsible for sex differences in CVD and atherosclerosis remain largely unknown. Sex hormones, sex chromosomes, and many genetic or epigenetic factors have been proposed to contribute to sex difference in CVD (Nour et al., 2023; Sakkers et al., 2023; Vaura et al., 2022). For example, sex chromosomes may influence atherosclerosis through sex-specific gene regulations (Sakkers et al., 2023; Vaura et al., 2022). It has also been well-established that estrogen has protective effects against atherosclerosis in animals and humans (Arnal et al., 2010; Billon-Gales et al., 2011; Hodgin et al., 2001; Nathan and Chaudhuri 1997). Thus, it is plausible MP exposure may interfere with those factors and only elicit observable atherosclerosis phenotypes in the lean male LDLR^−/−^ mice. For example, the protective effects of estrogen in female mice may render them to resistant to MP-induced atherosclerosis. Sexual dimorphic responses to various environmental exposures are still an understudied research topic. Many animal studies including the recent atherosclerosis studies only used one sex to investigate the atherogenic effects of MPs or NPs (Wang et al., 2023a; Wen et al., 2024a; Yang et al., 2025; Zhao et al., 2024; Zhong et al., 2024). Thus, it is important to include both sexes to investigate the potential impact of environmental factor exposures in animal models. While we observed sex-specific effects of MPs on atherogenesis in our study, the underlying mechanisms, potentially including the roles of sex hormones, sex chromosomes, and other factors, remain unclear. Further studies are needed to elucidate the mechanisms by which MPs and other environmental factors exert sex-specific influences on atherogenesis and CVD.

Previous in vitro studies have shown that MPs/NPs can induce cellular dysfunctions for macrophages, ECs, and SMCs, key cell types regulating atherosclerosis development (Prattichizzo et al., 2024). For example, MP exposure has been shown to trigger endocytosis and to elevate pro-inflammatory cytokine expression in monocytes/macrophages in vitro (Adler et al., 2024; Hirt and Body-Malapel 2020; Merkley et al., 2022). Exposure to NPs promoted cell migration and phenotypic switching in SMCs (Lomonaco et al., 2024; Persiani et al., 2025). In ECs, NP exposure activated inflammatory pathways and increased endothelial permeability with an increased oxidative stress (Lee et al., 2021; Vlacil et al., 2021). Atherosclerosis is a complex disease marked by cellular transdifferentiating and dynamic interactions among diverse vascular and immune cell types (Kong et al., 2022). To understand how MP exposures affect these different cells to induce atherosclerosis in vivo, we conducted scRNA-seq analysis of aorta. scRNA-seq enables high-resolution profiling of cellular identities and transcriptomic landscapes. The application of scRNA-seq to atherosclerosis research has uncovered shifts in cellular heterogeneity, offering novel insights into disease pathogenesis (Cui et al., 2023; Mocci et al., 2024; Mosquera et al., 2023). Our scRNA-seq results showed that MP exposure did not affect macrophage and monocyte populations but caused a reduction in B cells and T cells in the aorta. Previous studies also found that MP exposure led to significant reduction in donor-derived T cells and B cells but not myeloid cells in primary and secondary recipient mice (Jiang et al., 2024). Interestingly, exposure to MPs led to increased cell clusters including ECs, SMC-macrophages, stressed SMCs, and oligodendrocytes. MP exposure has been shown to induce the phenotypic changes and migration properties in SMCs (Zhong et al., 2024), which may correlate to our SMC results of scRNA-seq analysis. It is intriguing that MP exposure also increased the population of oligodendrocytes which are mainly expressed in central nervous system but not cardiovascular systems. Oligodendrocytes synthesize myelin to protect axons and other nerve fibers (Bradl and Lassmann 2010). However, aorta-derived mesoangioblasts can differentiate into oligodendrocytes (Wang et al., 2012), and MP exposure may affect aortic stem cell differentiation, leading to increased oligodendrocytes. Future studies are needed to investigate the impact of MP/NP exposure on stem cell differentiation in different tissues and the potential impact on disease development.

ECs are the key cell types mediating the initiation and progression of atherosclerosis. MPs have been shown to stimulate the expression of inflammatory cytokines and vascular cell adhesion molecules in ECs (Vlacil et al., 2021). Another study also found that MP exposure led to elevated ROS and cytotoxicity in human vascular ECs (Chen et al., 2023). Intriguingly, our RF-MP distribution results showed that MPs can be detected in the intima of vascular tissue and some apparently attached to the endothelia layer. scRNA-seq results indicated MP exposure affected EC population and different EC subclusters in the aorta samples. MP exposure strongly induced the proportion of EC subcluster cluster 3, macrophage-like ECs which highly expressed *Cd36* and *Fabp4*. CD36, a cell surface scavenger receptor, plays an important role in mediating lipid transport not only in immune cells but also the aortic ECs (Kalluri et al., 2019; Tian et al., 2020). Endothelial CD36 can enhance the oxidized-LDL uptake, leading to increased atherosclerosis (Tian et al., 2020; Wen et al., 2024b). FABP4, the fatty acid binding protein 4, is responsible for lipid metabolism in different cell types (Furuhashi et al., 2014). In human coronary ECs, FABP4 can increase the production of inflammatory cytokines and decrease the phosphorylation of eNOS (Fuseya et al., 2017; Wu et al., 2020). Our in vitro studies then found that MP treatment stimulated the expression of *Cd36* and *Fabp4* in murine primary ECs and human HMEC-1 ECs. MP exposure also elevated the expression of several other well-established pro-atherogenic genes such as *Il-6* in ECs. Further, MP exposure also led to increased production of ROS in human ECs which was consistent with previous studies (Chen et al., 2023). Thus, MP-stimulated pro-atherogenic gene expression may lead to endothelial dysfunctions and atherosclerosis development in those mice.

In summary, we investigated the impact of exposure to MPs on atherosclerosis development in lean male and female LDLR^−/−^ mice using an environmentally relevant dose and revealed that MP exposure induced sex-specific atherogenic effects in LDLR^−/−^ mice. Exposure to MPs led to significantly increased atherosclerosis in male but not female LDLR^−/−^ mice without altering adiposity and circulating lipid profiles. scRNA-seq analysis uncovered that exposure to MPs affected aortic cell proportions several key cell types mediating atherogenesis. It is particular interesting that MP exposure affected aortic EC subclusters and the related pro-atherogenic gene expression. In vitro studies showed the impact of MP exposure on the expression of several pro-atherogenic genes in murine and human ECs. Our results indicated the sex-specific atherogenic effects of MPs in a suitable laboratory animal model. Findings from this study contribute to our understanding of the association between MP exposure and increased CVD risk in humans.

## Supplementary Material

1

## Data Availability

Single cell RNA sequencing data have been deposited in the GEO database under accession code: GSE306246. Other Data will be made available on reasonable requests.

## Appendix A. Supplementary data

Supplementary data to this article can be found online at https://doi.org/10.1016/j.envint.2025.109938.
