# Supplementary material for: Microplastic exposure elicits sex-specific atherosclerosis development in lean low-density lipoprotein receptor-deficient mice
Source: Environ Int. Author manuscript; Available in PMC 2026 Jul 1. (PMC13321909; doi:10.1016/j.envint.2025.109938)
Supplement: 1 [file NIHMS2186743-supplement-1.pdf]

Supplementary Material for

Microplastic exposure elicits sex-specific atherosclerosis development in lean low-density lipoprotein receptor-deficient mice

Ting-An Lin,[1, 2] Jianfei Pan,[1] Mya Nguyen,[1] Qianyi Ma,[3] Liang Sun,[3] Sijie Tang,[1] Matthew J. Campen,[5] Hong Chen,[4] and Changcheng Zhou[1, 2, *]

[1]Division of Biomedical Sciences, School of Medicine, University of California, Riverside, CA, USA

[2]Environmental Toxicology Graduate Program, University of California, Riverside, CA, USA

[3]Research Computing, Department of Information Technology, Boston Children's Hospital, Boston, MA, USA

[4]Vascular Biology Program, Boston Children's Hospital and Harvard Medical School, Boston, MA, USA

[5]Department of Pharmaceutical Sciences, College of Pharmacy, University of New Mexico Health Sciences, Albuquerque, NM, USA

Summary of supplementary material:

Supplemental Figure 1-2

Supplemental Tables 1-4

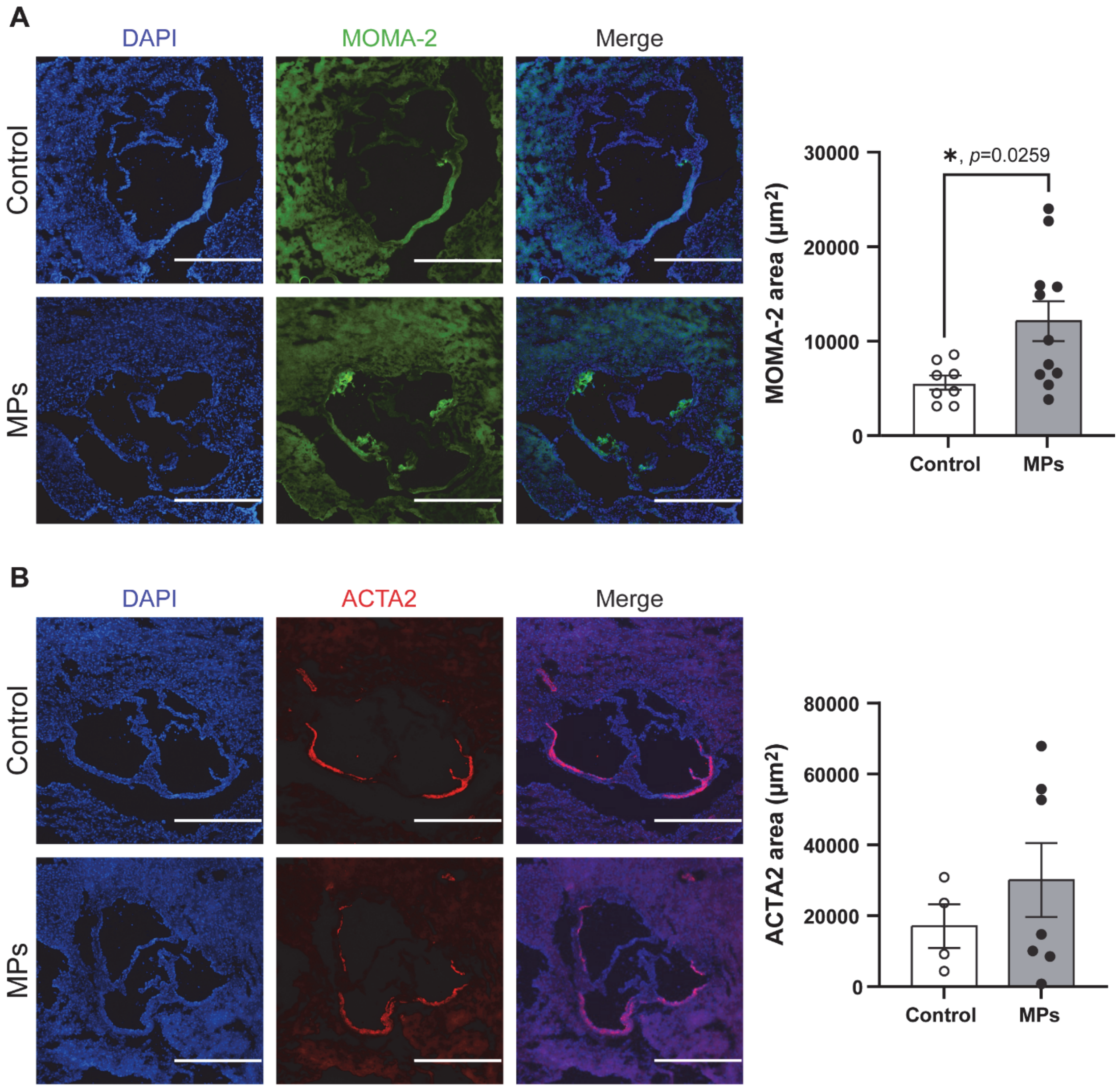

Supplemental Figure 1. Exposure to microplastics elicits macrophage accumulation in atherosclerotic plaques of LDLR$^{-/-}$ mice.

Four-week-old male LDLR$^{-/-}$ mice were fed a low-fat AIN76 diet and treated with 10 mg/kg body weight MPs or vehicle control daily by oral gavage daily for 9 weeks before euthanasia. Representative images of immunofluorescence staining of macrophage marker, MOMA-2 (A) and smooth muscle cell marker, ACTA2 (B) at the aortic root of male LDLR$^{-/-}$ mice (scale bar = 500 μm). The nuclei were stained with DAPI (blue). Quantification analysis of staining areas is displayed as indicated (n = 4-11; * $p < 0.05$; two-sample, two-tailed Mann-Whitney test). All data are presented as mean ± SEM.

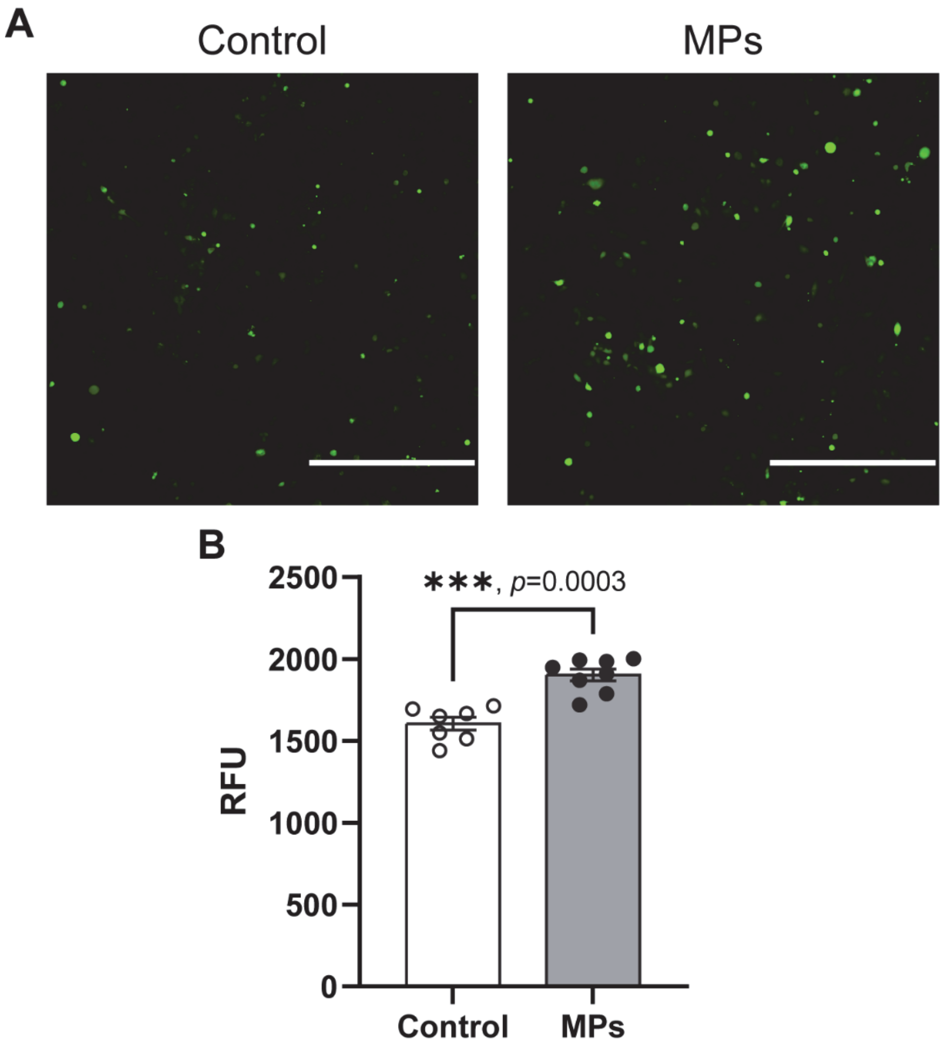

Supplemental Figure 2. Exposure to microplastics induces the production of reactive oxygen species in human endothelial cells in vitro.
(A) Human HMEC-1 endothelial cells were treated with vehicle control or 10 mg/L MPs for 24 hours followed by H_2DCFDA staining. (B) Quantification analysis of fluorescence (excitation = 495 nm; emission = 527 nm) is displayed as relative fluorescent unit, RFU (n = 8; *** p < 0.001; two-sample, two-tailed Mann-Whitney U test). All data are presented as mean ± SEM.

Supplemental Table 1. Primer sequences for QPCR.

Gene name	Sequence
Mouse *Gapdh*	F: AACTTTGGCATTGTGGAAGG
	R: GGATGCAGGGATGATGTTCT
Mouse *Cd36*	F: CAGTCGGAGACATGCT
	R: CTCGGGGTCCTGAGTT
Mouse *Fabp4*	F: ATGAAATCACCGCAGACGACAGGA
	R: TGTGGTCGACTTTCCATCCCACTT
Mouse *Ikkβ*	F: GAGCTCAGCCCAAAGAACAG
	R: AGGTTCTGCATCCCCTCTGG
Mouse *Il-1α*	F: GCACCTTACACCTACCAGAGT
	R: TGCAGGTCATTTAACCAAGTGG
Mouse *Il-1β*	F: GCAACTGTTCCTGAACTCAACT
	R: ATCTTTTGGGGTCCGTCAACT
Mouse *Il-6*	F: ACAAAGCCAGAGTCCTTCAGAGAG
	R: TTGGTCCTTAGCCACTCCTTC
Mouse *Mcp-1*	F: TTAAAAACCTGGATCGGAACCAA
	R: GCATTAGCTTCAGATTTACGGGT
Mouse *Icam-1*	F: GTGATCCCTGGGCCTGGTG
	R: GGAAACGAATACACGGTGATGG
Mouse *Vcam-1*	F: TACCAGCTCCCAAAATCCTG
	R: TCTGCTAATTCCAGCCTCGT
Human *Gapdh*	F: GGCCTCCAAGGAGTAAGACC
	R: AGGGGAGATTCAGTGTGGTG
Human *Cd36*	F: GCAAAATCCACAGGAAGTGATG
	R: GCTAGAAAACGAACTCTGTACGTATAAGG
Human *Fabp4*	F: ACTGGGCCAGGAATTTGACG
	R: CTCGTGGAAGTGACGCCTT
Human *Ikkβ*	F: ATCCCCGATAAGCCTGCCA
	R: CTTGGGCTCTTGAAGGATACAG
Human *Il-1α*	F: AGATGCCTGAGATACCCAAAACC
	R: CCAAGCACACCCAGTAGTCT
Human *Il-1β*	F: CGCCAATGACTCAGAGGAAGA
	R: AGGGCGTCATTCAGGATGAA
Human *Il-6*	F: GGTACATCCTCGACGGCATCT
	R: GTGCCTCTTTGCTGCTTTCAC
Human *Mcp-1*	F: GATGCAATCAATGCCCCAGTC
	R: TCCTTGGCCACAATGGTCTTG
Human *Icam-1*	F: ATGCCCAGACATCTGTGTCC
	R: GGGGTCTCTATGCCCAACAA
Human *Vcam-1*	F: TTTGACAGGCTGGAGATAGACT
	R: TCAATGTGTAATTTAGCTCGGCA

Supplemental Table 2. Quantification of atherosclerotic lesion sizes of LDLR$^{-/-}$ mice.

Group			n	Lesion area (μm^2)	*p*-value
Males	Aortic root	Control	10	17,157 ± 2983	0.0185
		MPs	10	27,979 ± 2678	
	BCA	Control	8	226.8 ± 60.17	0.0541
		MPs	7	1,643 ± 658.9	
Females	Aortic root	Control	12	38,341 ± 5713	0.1229
		MPs	10	4,9096 ± 5385	
	BCA	Control	7	957.2 ± 456.8	>0.9999
		MPs	7	1,222 ± 813.6	

Supplemental Table 3. Highly expressed genes in three different aortic EC subclusters.

Gene	*p*-value	log2 FC	pct.1	pct.2	Adj_ *p*-value	Subcluster
Klk10	2.49E-60	2.724032	0.757	0.165	8.04E-56	1
F2rl1	7.27E-57	2.794929	0.647	0.086	2.35E-52	1
Plek2	8.16E-55	2.883439	0.584	0.058	2.64E-50	1
Rhpn2	1.91E-53	2.690825	0.634	0.11	6.19E-49	1
Kcnn3	2.27E-48	2.854145	0.515	0.042	7.34E-44	1
Ocln	4.26E-40	2.852135	0.467	0.055	1.38E-35	1
Klk11	4.82E-35	3.281635	0.411	0.042	1.56E-30	1
Tnfrsf26	3.79E-33	2.662938	0.434	0.063	1.23E-28	1
Unc13d	1.69E-32	2.834465	0.373	0.029	5.47E-28	1
Elovl7	6.82E-31	2.7891	0.411	0.065	2.21E-26	1
Pla2g5	2.18E-27	3.135782	0.328	0.029	7.07E-23	1
Degs2	6.21E-25	2.698205	0.328	0.039	2.01E-20	1
Pdzk1ip1	2.27E-24	2.953048	0.29	0.024	7.33E-20	1
Lpcat2	1.83E-19	2.853882	0.229	0.016	5.91E-15	1
Adra2a	1.64E-17	2.826551	0.279	0.055	5.32E-13	1
Gdf7	1.72E-16	2.952961	0.2	0.016	5.58E-12	1
Pak4	2.45E-16	2.832144	0.247	0.045	7.94E-12	1
Kcnh1	2.39E-15	2.829778	0.213	0.029	7.74E-11	1
Rorc	3.86E-15	2.690321	0.225	0.037	1.25E-10	1
Nkx2-3	4.63E-14	2.689159	0.184	0.021	1.5E-09	1
Acpp	9.55E-14	2.932178	0.187	0.024	3.09E-09	1
Dkk2	8.18E-12	4.058169	0.139	0.01	2.65E-07	1
Coch	2E-11	3.423119	0.157	0.021	6.47E-07	1
Dcdc2a	6.25E-11	3.531043	0.119	0.005	2.02E-06	1
Ifi27l2b	1.04E-10	3.105028	0.139	0.016	3.35E-06	1
Fzd9	4.96E-09	3.167268	0.13	0.021	0.00016	1
Nfe2	1.83E-08	4.185404	0.092	0.005	0.000592	1
Cdca7l	2.04E-08	4.702718	0.092	0.005	0.000658	1
Kcne3	5.57E-08	2.957763	0.088	0.005	0.001802	1
Klk13	6.99E-08	3.108092	0.092	0.008	0.002261	1
Etnk2	1.35E-07	2.71821	0.106	0.016	0.004367	1
Lrtm2	2.9E-07	3.497946	0.085	0.008	0.009389	1
H2-DMb1	2.91E-07	2.92369	0.097	0.013	0.009412	1
Kcnk10	3.76E-07	4.156051	0.072	0.003	0.012145	1
Hoxa10	6.25E-07	4.253783	0.063	0	0.020213	1
Cacna1a	7.32E-07	2.931646	0.097	0.016	0.023674	1
Frmd5	5.42E-06	3.485958	0.076	0.01	0.175305	1
Sdsl	6.41E-06	3.209682	0.081	0.013	0.207209	1
Psma8	1.07E-05	3.777184	0.056	0.003	0.346779	1

Raet1d	2.33E-05	3.500729	0.058	0.005	0.754614	1
Fam187b	4.16E-05	2.772725	0.056	0.005	1	1
Bco1	9.67E-05	3.145851	0.052	0.005	1	1
Abcc3	0.000155	3.163612	0.049	0.005	1	1
Vil1	0.000242	3.543676	0.047	0.005	1	1
Cfi	0.000477	3.316424	0.031	0	1	1
Klk7	0.00192	3.105561	0.031	0.003	1	1
E2f2	0.002421	3.215333	0.036	0.005	1	1
Pla2g4f	0.002477	3.291043	0.036	0.005	1	1
Rnf133	0.00306	3.324647	0.029	0.003	1	1
Fgr	0.005048	2.839697	0.027	0.003	1	1
Vtn	3.55E-19	6.737952	0.155	0.007	1.15E-14	2
Ebf2	2.17E-17	5.710497	0.206	0.032	7.02E-13	2
Ildr2	2.79E-14	6.17347	0.134	0.012	9.02E-10	2
Thbs4	4.37E-12	9.286775	0.092	0.003	1.41E-07	2
Cldn1	2.55E-11	8.676136	0.092	0.005	8.23E-07	2
Tenm2	5.8E-11	7.160916	0.071	0	1.88E-06	2
Clstn2	5.87E-10	5.792527	0.092	0.008	1.9E-05	2
H2-M9	2.07E-09	5.709073	0.088	0.008	6.7E-05	2
Sfrp5	4.56E-09	8.06711	0.076	0.005	0.000148	2
Apod	8.72E-09	8.442288	0.202	0.073	0.000282	2
Krt19	2.46E-08	7.174487	0.059	0.002	0.000797	2
Cdkn2b	4.13E-08	5.90307	0.05	0	0.001336	2
Lypd2	4.13E-08	8.193395	0.05	0	0.001336	2
Clic6	4.13E-08	6.708601	0.05	0	0.001336	2
Ngfr	8.97E-08	5.731557	0.055	0.002	0.002902	2
Mpzl2	1.49E-07	6.559548	0.059	0.003	0.004819	2
Cyp2s1	1.53E-07	6.993653	0.046	0	0.004961	2
Msln	1.53E-07	8.4235	0.046	0	0.004961	2
Elmod1	3.39E-07	6.011175	0.05	0.002	0.010979	2
Ctss	7.49E-07	6.619494	0.059	0.005	0.024216	2
Cdk15	2.12E-06	5.659999	0.038	0	0.068624	2
Lrrn4	2.12E-06	8.094025	0.038	0	0.068624	2
Il17re	2.12E-06	5.770397	0.038	0	0.068624	2
Wnt6	2.95E-06	6.887753	0.059	0.007	0.095351	2
Ccl11	3.75E-06	5.757307	0.126	0.042	0.121185	2
Myoc	7.11E-06	8.149008	0.046	0.003	0.22983	2
Pax1	7.91E-06	6.531967	0.034	0	0.255982	2
Wnt10a	1.66E-05	5.718803	0.038	0.002	0.536657	2
Il18r1	2.96E-05	6.117791	0.029	0	0.958353	2
Fcgr3	2.96E-05	6.878706	0.029	0	0.958353	2

Slc6a13	2.96E-05	6.237991	0.029	0	0.958353	2
Svopl	6.1E-05	5.947596	0.034	0.002	1	2
Plet1	6.1E-05	5.859528	0.034	0.002	1	2
Tmem273	8.75E-05	6.020397	0.038	0.003	1	2
Upk3b	0.000112	10.1514	0.025	0	1	2
Muc16	0.000112	6.01898	0.025	0	1	2
Kcnj13	0.000224	5.792807	0.029	0.002	1	2
Htr7	0.000224	5.69004	0.029	0.002	1	2
Col6a5	0.000226	6.679424	0.029	0.002	1	2
Vmn1r160	0.000423	6.307396	0.021	0	1	2
Fxyd3	0.000423	5.864665	0.021	0	1	2
Chst4	0.000423	6.590894	0.021	0	1	2
Galr1	0.000423	6.113234	0.021	0	1	2
Grm3	0.000824	6.501193	0.025	0.002	1	2
Lrp2	0.001629	5.932433	0.017	0	1	2
Upk1b	0.003027	7.136718	0.021	0.002	1	2
Hcn4	0.004984	5.95074	0.042	0.012	1	2
Btbd35f18	0.006401	5.803245	0.013	0	1	2
Btbd35f28	0.006401	6.217731	0.013	0	1	2
Btbd35f25	0.006401	5.784881	0.013	0	1	2
Cd300lg	2.3E-128	6.375101	0.882	0.041	7.4E-124	3
Gpihbp1	6.6E-128	6.293514	0.903	0.048	2.1E-123	3
Pde2a	1.8E-119	5.832061	0.91	0.075	5.7E-115	3
Cd36	4.8E-110	5.518469	0.951	0.13	1.6E-105	3
Aqp7	1.5E-108	6.756056	0.75	0.029	4.9E-104	3
Kdr	3.8E-101	5.331241	0.889	0.111	1.24E-96	3
Ablim3	4E-101	5.25076	0.785	0.054	1.3E-96	3
Robo4	2.5E-100	5.023118	0.882	0.111	8.09E-96	3
Ccdc85a	7.2E-99	5.733669	0.743	0.042	2.33E-94	3
Car4	1.45E-92	6.054053	0.667	0.028	4.69E-88	3
Arhgap27	8.24E-92	5.048769	0.806	0.088	2.66E-87	3
Tcf15	8.52E-92	6.006525	0.667	0.029	2.76E-87	3
Btnl9	3.74E-91	6.374118	0.625	0.018	1.21E-86	3
Rgcc	1.16E-90	5.75034	0.806	0.094	3.76E-86	3
Rbp7	6.71E-83	7.28084	0.549	0.01	2.17E-78	3
Depp1	1.21E-82	6.112876	0.66	0.044	3.92E-78	3
Fscn1	3.52E-82	5.235367	0.618	0.029	1.14E-77	3
Kcna5	8.07E-80	5.28811	0.611	0.031	2.61E-75	3
Slc28a2	9.57E-68	5.560813	0.5	0.019	3.09E-63	3
Csf2rb	1.44E-65	5.620867	0.458	0.012	4.67E-61	3
Cdh23	2.28E-56	5.621654	0.417	0.015	7.36E-52	3

Rsad2	3.02E-48	5.482516	0.424	0.031	9.76E-44	3
Gpr160	2.77E-45	5.031784	0.333	0.01	8.95E-41	3
Slc26a10	1.22E-44	6.843598	0.299	0.004	3.96E-40	3
Slc1a1	4.34E-39	8.028206	0.264	0.004	1.4E-34	3
Lhx6	2.78E-36	5.155209	0.257	0.006	8.98E-32	3
Pcdh17	1.69E-33	6.959213	0.222	0.003	5.48E-29	3
Fabp9	5E-32	6.162813	0.278	0.018	1.62E-27	3
Nepn	7.7E-26	6.784849	0.167	0.001	2.49E-21	3
Lrrc36	2.08E-21	5.278597	0.174	0.009	6.71E-17	3
Slc22a23	3.82E-16	5.088961	0.139	0.009	1.24E-11	3
Srarp	2.6E-15	6.946114	0.09	0	8.4E-11	3
Nr5a2	3.11E-14	6.305589	0.083	0	1E-09	3
Stc1	5.01E-14	6.539539	0.153	0.018	1.62E-09	3
Krt222	3.7E-13	6.482871	0.076	0	1.2E-08	3
Hrh2	3.7E-13	6.291247	0.076	0	1.2E-08	3
Ackr1	8.27E-11	5.083004	0.09	0.006	2.67E-06	3
Mmrn1	1.01E-09	8.101776	0.076	0.004	3.26E-05	3
Htr2b	7.39E-09	5.648363	0.049	0	0.000239	3
Ifi208	7.39E-09	5.335251	0.049	0	0.000239	3
Duox2	1.27E-08	6.213614	0.056	0.001	0.000412	3
Sez6l2	1.38E-08	5.022891	0.062	0.003	0.000446	3
Lzts1	1.43E-07	5.79953	0.056	0.003	0.004636	3
Nts	1.05E-06	5.388999	0.056	0.004	0.034006	3
Scgb3a1	1.06E-06	5.87334	0.035	0	0.034276	3
Glp1r	1.06E-06	5.693281	0.035	0	0.034276	3
Lipg	1.55E-06	5.852723	0.049	0.003	0.05028	3
Reln	1.02E-05	5.129832	0.049	0.004	0.329253	3
Cym	1.29E-05	5.735985	0.028	0	0.416789	3
Dtx1	0.000335	5.151116	0.069	0.018	1	3

Supplemental Table 4. Top DEGs of aortic EC subclusters in pseudotime heatmap.

Gene	Subcluster	*p*-value	*q*-value	Gene	Subcluster	*p*-value	*q*-value
Pecam1	1	3.14E-226	2.48E-223	*Myh11*	2	0	0
Ehd4	1	5.13E-162	2.27E-159	*Acta2*	2	0	0
Fam3c	1	5.17E-160	2.26E-157	*mt-Co2*	2	4.89E-136	1.65E-133
Apoe	1	1.59E-158	6.76E-156	*mt-Atp6*	2	1.18E-143	4.33E-141
Heg1	1	2.24E-172	1.10E-169	*mt-Co3*	2	2.04E-151	8.15E-149
Tm4sf1	1	2.85E-180	1.59E-177	*Smoc2*	2	3.29E-150	1.28E-147
Ltbp4	1	2.34E-156	9.82E-154	*Rarres2*	2	1.54E-159	6.62E-157
Cd24a	1	2.66E-178	1.46E-175	*Igfbp6*	2	0	0
Gja5	1	2.30E-145	8.63E-143	*Igfbp7*	2	3.48E-321	5.37E-318
Tmem158	1	4.07E-134	1.33E-131	*Rgs5*	2	0	0
Bmp4	1	2.02E-176	1.09E-173	*Sparcl1*	2	6.88E-247	6.36E-244
Cgnl1	1	5.48E-140	1.93E-137	*Gpx3*	2	6.55E-188	3.85E-185
Cfh	1	4.08E-175	2.13E-172	*Gpc3*	2	6.44E-145	2.39E-142
Eln	1	2.48E-198	1.51E-195	*Apod*	2	0	0
Klk10	1	9.73E-247	8.74E-244	*Thbs4*	2	1.64E-150	6.46E-148
Sfrp1	1	0.00E+00	6.87E-306	*Sfrp5*	2	4.81E-201	3.05E-198
Vcam1	1	8.50E-163	3.87E-160	*Aqp1*	3	3.06E-239	2.60E-236
Atp2a3	1	1.54E-134	5.09E-132	*Slfn5*	3	6.39E-155	2.62E-152
Krt80	1	1.19E-314	1.68E-311	*Adgrf5*	3	6.75E-229	5.60E-226
Ace	1	1.22E-223	9.40E-221	*Pde2a*	3	0	0
Ptprj	1	1.60E-204	1.06E-201	*Robo4*	3	2.03E-162	9.14E-160
Ctsh	1	1.58E-173	7.99E-171	*Scarb1*	3	2.43E-284	2.54E-281
Cytl1	1	0	0	*Rbp7*	3	2.10E-209	1.42E-206
Thbs1	1	2.23E-181	1.26E-178	*Depp1*	3	4.34E-223	3.26E-220
Clu	1	0	0	*Btnl9*	3	1.18E-192	7.04E-190
Igfbp5	2	4.54E-308	5.87E-305	*Cdkn1a*	3	1.02E-134	3.39E-132
C3	2	4.66E-170	2.25E-167	*Sgk1*	3	3.67E-173	1.83E-170
Ccn2	2	2.41E-226	1.95E-223	*Adgrl4*	3	2.88E-318	4.24E-315
Flna	2	9.73E-156	4.04E-153	*Flt1*	3	7.08E-287	7.63E-284
Ogn	2	6.01E-165	2.86E-162	*Fabp4*	3	0	0
Lmod1	2	1.46E-140	5.21E-138	*Cd36*	3	0	0
Cnn1	2	6.30E-163	2.91E-160	*Ucp2*	3	1.02E-164	4.77E-162
Col6a2	2	6.87E-174	3.53E-171	*Xdh*	3	1.02E-293	1.14E-290
Hspb7	2	3.16E-141	1.14E-138	*Col4a2*	3	6.06E-134	1.96E-131
Lrp1	2	1.51E-147	5.73E-145	*Lpl*	3	0	0
Dcn	2	0	0	*Timp4*	3	4.95E-302	5.93E-299
Gsn	2	0	0	*Ablim3*	3	7.56E-240	6.61E-237
Col1a2	2	1.12E-212	7.69E-210	*Itga6*	3	4.20E-150	1.62E-147

Mylk	2	9.53E-140	3.28E-137	*Cd300lg*	3	0	0
Postn	2	4.54E-220	3.34E-217	*Gpihbp1*	3	0	0
Col6a1	2	5.64E-140	1.96E-137	*Car4*	3	0	0
Tgfbi	2	9.72E-154	3.93E-151	*Ccdc85a*	3	2.56E-175	1.36E-172
Col3a1	2	0	0	*Rgcc*	3	0	0
Col1a1	2	0	0	*Pcdh1*	3	3.01E-138	1.02E-135
Ccn3	2	7.79E-213	5.48E-210	*Kdr*	3	3.92E-266	3.84E-263
Myl9	2	1.84E-303	2.28E-300	*C1qtnf9*	3	1.21E-184	6.98E-182
Plin4	2	7.17E-143	2.61E-140	*Mgll*	3	8.88E-268	8.97E-265
Tagln	2	1.37E-296	1.58E-293	*Tcim*	3	3.61E-201	2.34E-198
Itga8	2	3.08E-215	2.22E-212	*Aqp7*	3	0	0
Tpm2	2	3.73E-256	3.55E-253	*Mcf2l*	3	1.06E-200	6.59E-198
